# Magnitude of wasting and underweight among children 6–59 months of age in Sodo Zuria District, South Ethiopia: a community based cross-sectional study

**DOI:** 10.1186/s13104-018-3880-x

**Published:** 2018-11-03

**Authors:** Efrata Girma Tufa, Samson Kastro Dake, Eyasu Tamiru Bekru, Habtamu Azene Tekle, Tesfahun Molla Bobe, Banchalem Nega Angore, Fithamlak Bisetegen Solomon

**Affiliations:** 1 Department of Reproductive Health and Nutrition, School of Public Health, College of Health Science and Medicine, Wolaita Sodo University, Sodo, Ethiopia; 2 Department of Nursing, College of Health Science and Medicine, Wolaita Sodo University, Sodo, Ethiopia; 3 School of Medicine, College of Health Science and Medicine, Wolaita Sodo University, Sodo, Ethiopia; 4 Department of Midwifery, College of Health Science and Medicine, Wolaita Sodo University, Sodo, Ethiopia; 5 Department of Medical Laboratory, School of Medicine, College of Health Science and Medicine, Wolaita Sodo University, Sodo, Ethiopia

**Keywords:** Children, 6–59 months, Undernutrition, Wasting and underweight

## Abstract

**Objectives:**

The study aimed to determine the prevalence of wasting and underweight, and identify associated factors in Sodo Zuria district in South Ethiopia.

**Results:**

The prevalence of wasting and underweight were 11.1% and 14.0%, respectively. Wasting was significantly associated with male gender, diarrheal morbidity 2 weeks prior to the study and early initiation of complementary feeding. Predictors of underweight were diarrheal morbidity 2 weeks prior to the study and paternal illiteracy. The prevalence of wasting and underweight among under-five children is common in the study area. Diarrheal morbidity was associated with both wasting and underweight. Interventions targeting prevention of diarrheal morbidity through hygienic practices and creating awareness on infant feeding practices need to be implemented in the study area.

**Electronic supplementary material:**

The online version of this article (10.1186/s13104-018-3880-x) contains supplementary material, which is available to authorized users.

## Introduction

Globally, at least one in three people experience malnutrition in some form [1] and the prevalence of child undernutrition is high in low- and middle-income countries [2]. Despite the decreasing trend in the prevalence, undernutrition continued to be a public health problem in developing countries [3]. The number of people without access to adequate calories in the world has increased since 2015 [4].

The number of undernourished people globally rose from 777 million in 2015 to 815 million in 2016 [5]. Globally, fifty-one million children were wasted and 16 million were underweight in 2017. Africa and Asia bear the greatest share of all forms of malnutrition and in Eastern Africa the prevalence of wasting is 6% [6]. In Ethiopia, according to the Ethiopian Demographic and Health Survey (EDHS) of 2016, the prevalence of wasting and underweight were 10% and 24%, respectively [7]. The prevalence of wasting changed little (from 12 to 10%) in 16 years period nationally. However, the prevalence of underweight consistently decreased from 41 to 24% [7, 8]. Wasting and underweight prevalence of Southern Ethiopia were 6 and 21.1%, respectively [7].

About one-third of deaths among children below 5 years of age were attributed to undernutrition and it can lead children to be at greater risk of death and severe illness due to common childhood infections [9]. Undernutrition in children leads to physical and mental impairment [10–14].

The global progress to reduce malnutrition is not rapid enough to meet internationally agreed targets, including Sustainable Development Goal (SDG) target to end all forms of malnutrition by 2030. Improving nutrition will be a catalyst for achieving other SDGs. Despite few studies were done at national and regional levels, the prevalence and risk factors at sub-regional or community level have been insufficiently emphasized. So this study tries to assess the burden of wasting and underweight among children aged 6–59 month at Sodo Zuria district, South Ethiopia.

## Main text

### Methods

#### Study setting

A community based cross sectional study was conducted among children aged 6–59 months paired with mothers/caretakers. Data was collected from May to June 2017. The study area is located at 380 km south of Addis Ababa. Sodo Zuria district is administratively structured into 30 kebeles (lowest administrative units) and has a census projected total population of 394, 772; 24,647 were children in the age range of 6–59 months.

#### Population and sampling

All randomly selected children 6–59 months of age paired with their mothers/caregivers were the study population. Sample size was calculated with an expected prevalence of 36.6% [7], 95% confidence level, 5% margin of error and 5% non response rate. The total sample size calculated for this study was 375. Out of 30 kebeles, 4 kebeles were selected randomly. The total sample size was allocated to each kebele proportionally. Households were selected using systematic random sampling and for households with more than one child in the age range of 6–59 months, one child was selected randomly.

#### Data collection

Structured questionnaire was used and mothers/caregivers were interviewed face to face. Five bachelor degree graduates with prior experience on data collection and fluent speakers of the local language were recruited and intensively trained on the data collection questionnaire, selecting study participants, anthropometric measurements and ethics. They were also standardized on taking anthropometric measurements. The questionnaire was pretested on 5% of the sample size in a community other than the study area and revised. The length of a child (aged 6–23 months) was measured with a horizontal wooden length board in recumbent position. The height of a child (aged 24–59 months) was measured with a vertical wooden height board while the child standing upright in the board. The length and height measurements were read to the nearest 0.1 cm. Weight was measured using seca digital weight scale and read to the nearest 0.1 kg. All measurements were taken twice and the mean was used for analysis. Supervisors checked the completeness and consistency of the questionnaire and errors were corrected on spot in the field.

#### Operational definitions

*Wasting* was computed when the weight-for-height z-score (WHZ) is below − 2 SD in the national center for Health Statistics (NCHS) growth curve.

*Severe wasting* was computed when the weight-for-height z-score (WHZ) is below − 3 SD in the NCHS growth curve.

*Underweight* was computed when the weight for age z-score (WAZ) is below − 2 SD in the NCHS growth curve.

*Severe underweight* was computed when the weight for age z-score (WAZ) is below − 3 SD in NCHS growth curve.

#### Data analysis

Data were entered into Epi Info software version 3.5.1 and exported to SPSS version 20 for analysis. WAZ data were analyzed using WHO Anthro software and compared using NCHS reference standard. Descriptive statistics was done for all variables. Bivariate analysis was performed to determine crude association and variables with p < 0.25 in bivariate analysis were considered candidate for multivariate analysis. Multivariate logistic regression analysis was conducted to control confounders and identify predictor variables. Statistical significance was considered at p < 0.05.

### Results

#### Sociodemographic characteristics

Out of 375 children, 342 participated in the actual study which gives a response rate of 91.2%. From all study participant mothers/caregivers, majority 299 (87.43%) were married, Wolaita in ethnic group 336 (98.25%) and protestant religion followers 246 (71.93%). One hundred sixty-four (47.95%) of the children were male and 104 (30.41%) were 6–23 months of age (Additional file 1: Table S1).

#### Obstetric, maternal and child morbidity related characteristics

Majority 272 (79.53%) of the children were born in the health facility and 229 (66.96%) were born at 9 months of gestational age. Three-fourth 257 (75.15%) of the children had a normal birth weight and 69 (20.17%) were born in < 24 months gap with the previous birth. (Additional file 2: Table S2).

#### Child caring practice and environmental health characteristics

Two-third 228 (66.67%) of the children were started on breastfeeding within 1 h after birth, 334 (97.66%) were given colostrums and 301 (88.01%) did not get pre lacteal foods or liquids. Of the children 195 (57.02%) started complementary food at 6 months of age, and 250 (73.10%) never ate meat in their life. (Additional file 3: Table S3).

#### Prevalence of wasting and underweight

Prevalence of wasting was 38 (11.1%), and severe wasting was 15 (4.4%). The highest prevalence of wasting (3.2%) was seen among children aged 48–59 months. The prevalence of underweight among study participants was 49 (14%) of which severe underweight accounts for 11 (3%). The highest prevalence of underweight (3.8%) was seen among children aged 48–59 months.

#### Predictors of wasting and underweight

As indicated in Table 1, The likelihood of being wasted was 60% higher for children who started breastfeeding between 1 and 24 h of birth than their counterparts (AOR = 1.6, 95% CI 1.2–2.3). Children who had diarrhea in the past 2 weeks were 2.8 times (AOR = 2.8, 95% CI 3.2–7.4) higher odds of being wasted than children had no diarrhea. Children who had diarrhea 2 weeks prior to this study were 2.8 times (AOR = 3.9, 95% CI 1.8–4.4) more likely to be underweight than children without diarrheal disease. Risk of underweight among children whose fathers were illiterate was 5.4 times (AOR = 6.7, 95% CI 2.5–9.1) more likely than children whose fathers were literate. Female children were 3.2 times (AOR = 2.5, 95% CI 1.5–4.1) more likely to be underweight than males (Table 1).

**Table 1:** Factors associated with wasting and underweight among children aged 6–59 months at Sodo Zuria district, Wolaita Zone, South Ethiopia, June 2017 (n = 342)

Explanatory variables	Under weight		COR (95% CI)	AOR (95% CI)
	Yes	No		
Child sex				
Male	9	124	1.0	1.0
Female	40	169	1.8 (1.3–2.7)*	1.6 (0.7–1.3)
Paternal formal education				
Yes	29	82	1.0	1.0
No	33	111	2.8 (2.3–3.9)*	5.4 (2.5–9.1)*
Ownership of livestock				
Yes	41	193	1.0	
No	8	100	1.4 (1.1–1.9)	
Place of delivery				
Health facility	39	233	1.0	
Home	10	60	0.6 (0.4–0.9)*	
Availability of latrine				
Yes	41	208	1.0	1.0
No	8	85	1.6 (2.1–4.5)	1.7 (0.5–1.6)
Fed pre-lactation feeding				
No	39	262	1.0	1.0
Yes	10	31	2.9 (2.3–6.1)	0.6 (0.9–2.6)
Diarrheal morbidity in the last 2 weeks				
No	29	224	1.0	1.0
Yes	20	175	2.3 (2.1–3.8)*	2.8 (1.8–4.4)*

Explanatory variables	Wasting		COR (95% CI)	AOR (95% CI)
	Yes	No		
Child sex				
Male	17	143	143	3.2 (1.9–5.3)*
Female	21	161	161	1.0
Age at complementary feeding started (in months)				
≥ 6	33	225	225	1.0
< 6	5	4	4	2.2 (1.8–3.9)*
Immunization				
Yes	34	293	293	1.0
No	4	11	11	1.7 (0.8–3.8)
Ever use of family planning				
Yes	18	152	152	1.0
No	20	152	152	1.7 (0.6–2.1)
Initiation of breast feeding				
Within 1 h of delivery	24	204	204	1.0
After 1 h of delivery	14	100	100	1.6 (0.7–5.3)
Pre-lactation feeding				
No	35	266	266	1.0
Yes	3	38	38	1.5 (0.9–2.4)
Availability of latrine				
Yes	20	239	239	1.0
No	18	75	75	1.5 (0.8–2.2)
Diarrheal morbidity in the last 2 weeks				
No	17	236	236	1.0
Yes	21	68	68	2.8 (2.2–7.4)*

*statistically significant at p<0.05

### Discussion

In this study, the prevalence of wasting was 11.1%, which is comparable with studies reported in Nigeria (9.5%) [15], Vietnam (10.2%) [16], South Asia (11%) [17], Ethiopia (8.9%) [18] and the 2016 EDHS report (10%) [7]. Higher prevalence levels were reported in Sudan [19] and Ethiopia [20]. However, it is higher than other studies reported in Bete Israel and Mongolia [21, 22]. The prevalence of underweight in this study was 14.0%. This is comparable with a study done in Bete Israel, Ethiopia (16.6%) [21]. But it is lower than studies reported elsewhere and in the country [7, 16, 17, 20, 23–26]. However, this is higher than a report in Mongolia (4.7%) [22].

In this study, the odd of being wasted was 3 times higher for boys as compared to girls. Many studies conducted in Ethiopia and other parts of the Globe have reported similar findings [10, 19, 25, 27, 28]. It is unlikely that gender preference and differential feeding be the reason since it is females who usually receive less food in Ethiopian culture. There might be a genetic basis for boy’s vulnerability since it is reported in many studies.

In this study, children who started complementary feeding before the age of 6 months had higher odds of being wasted when compared with children who started at the age of 6 months. This is in line with other study findings which revealed that children who exclusively breast fed for < 6 months were more likely to be wasted as compared with those exclusively breast fed for ≥ 6 months.

In the current study, parental formal educational status was a predictor of underweight. Compared with children of educated fathers, children born from uneducated fathers had higher odds of being underweight. Other studies reported similar finding [16, 29]. The possible explanation might be that education might influence father’s attitude towards child’s nutrition and educated fathers tend to invest in children’s feeding [16, 25, 29–31].

Presence of diarrheal morbidity 2 weeks prior to data collection was an independent predictor for both wasting and underweight. Similar finding was reported in Ethiopia and elsewhere [17, 25, 27]. The possible reason might be that diarrhea leads to loss of appetite leading to decreased food intake, malabsorption and finally malnutrition. On the other hand, malnutrition leads diarrhea. Since the current study is cross-sectional, it is difficult to conclude the direction of association.

### Conclusion and recommendation

A high proportion of wasting and underweight was found among children 6–59 months in Sodo Zuria district. Wasting was associated with male sex, diarrheal morbidity 2 weeks prior to the study and initiation of complementary feeding before 6 months, whereas underweight was found to be significantly associated with being a child of an uneducated father and diarrheal morbidity 2 weeks prior to the study. Efforts should be commenced towards prevention of diarrheal morbidity through hygiene practice and awareness creation on infant feeding practices and specifically on initiation of complementary feeding.

## Limitation of the study

Since it was a cross-sectional design, it was difficult to examine any potential temporal relationships. Certain level of recall bias was expected with regard to events happened in the past; such as history of illness and breastfeeding patterns immediately after birth and then after. We have no data for some biomedical indicators like arm circumference.

## Additional files

**Additional file 1: Table S1.** Socio-demographic characteristics of study participants in Sodo Zuria district, South Ethiopia, June 2017.

**Additional file 2: Table S2.** Obstetric, child morbidity and other maternal characteristics of study participants in Sodo Zuria district, South Ethiopia, June 2017.

**Additional file 3: Table S3.** Child caring practice and environmental health characteristics of study participants in Sodo Zuria district, South Ethiopia, June 2017.

## Abbreviations

AIDS: acquired immune deficiency syndrome
AOR: adjusted odds ratio
CI: confidence interval
COR: crude odds ratio
EDHS: Ethiopian Demographic and Health Survey
HIV: human immunodeficiency virus
NCHS: National Center for Health Statistics
SD: standard deviation
SDG: Sustainable Development Goals
SPSS: Statistical Package for Social Sciences
WAZ: weight-for-age z-score
WHO: World Health Organization
WHZ: weight-for-height z-score

## References

[1] Institute IFPR (2017). 2017 global food policy report.

[2] Black R, Allen LH, Bhutta ZA, Caulfield LE, Onis MD, Ezzati M (2008). Maternal and child undernutrition: global and regional exposures and health consequences. Lancet..

[3] UNICEF. 2017 famine response progress update. 2017. https://www.unicef.org/appeals/famine.html. Accessed 22 Aug 2017.

[4] FAO. World hunger on the rise again: reversing years of progress. 2017. http://www.fao.org/news/story/en/item/902489/icode/. Accessed 22 Aug 2017.

[5] Institute IFPR (2018). 2018 global food policy report.

[6] UNICEF, Organization WH, World T (2018). Levels and trends in child malnutrition: key findings of the 2018 edition of child malnutrition estimates.

[7] CSA, ICF (2016). Ethiopia demographic and health survey.

[8] Macro O, CSA (2000). Ethiopia demographic and health survey.

[9] UNICEF (2013). Improving child nutrition: the achievable imperative for global progress.

[10] Taylor L. From food crisis to nutrition: challenges and possibilities in Ethiopia’s nutrition sector. Analysing Nutrition Governance: Ethiopia Country Report. 2012.

[11] Abuya BA, Ciera JM, Kimani-Murage E (2012). Effect of mother’s education on child’s nutritional status in the slums of Nairobi. BMC Pediatr..

[12] Wondafrash M, Amsalu T, Woldie M (2012). Feeding styles of caregivers of children 6–23 months of age in Derashe special district, Southern Ethiopia. BMC Public Health..

[13] Dewey KG, Begum K (2011). Long-term consequences of stunting in early life. Matern Child Nutr..

[14] Yimer G (2000). Malnutrition among children in Southern Ethiopia: level and risk factors. Ethiop J Health Dev..

[15] Babatunde RO, Olagunju FI, Fakayode SB, Sola-Ojo FE (2011). Prevalence and determinants of malnutrition among under-five children of farming households in Kwara State, Nigeria. J Agric Sci..

[16] Siddiqi NA, Haque N, Goni MA (2011). Malnutrition of under-five children: evidence from Bangladesh. Asian J Med Sci..

[17] Sapkota VP, Gurung CK (2009). Prevalence and predictors of underweight, stunting and wasting in under-five children. Nepal Health Res Counc..

[18] Yalew BM (2014). Prevalence of malnutrition and associated factors among children age 6–59 months at Lalibela Town Administration, North WolloZone, Anrs, Northern Ethiopia. J Nutr Disord Ther..

[19] Ola E, Mofida A (2011). Nutritional status of the children under age of five in a decertified area of Sudan-Alrawakeeb valley. Int J Curr Res..

[20] Mengistu K, Alemu K, Destaw B (2013). Prevalence of malnutrition and associated factors among children aged 6–59 months at Hidabu Abote District, North Shewa, Oromia Regional State. J Nutr Disord Ther..

[21] Asres G, Eidelman AI (2011). Nutritional assessment of Ethiopian Beta-Israel children: a cross-sectional survey. Breastfeed Med..

[22] Otgonjargal D, Bradley A, Woodruff F, Batjarga L, Davaalkham D (2012). Nutritional status of under- five children in Mongolia. J Med Med Sci..

[23] Kwena AM, Terlouw DJ, de Vlas SJ, Phillips-Howard PA, Hawley WA (2003). Prevalence and severity of malnutrition in pre-school children in a rural area of western Kenya. Am J Trop Med Hyg.

[24] Edris M (2007). Assessment of nutritional status of preschool children of Gumbrit. Ethiop J Health Dev..

[25] Teshome B, Kogi- Makau W, Getahun Z, Taye G (2006). Magnitude and determinants of stunting in children under five years of age in food surplus region of west Gojam zone. Ethiop J Health Dev..

[26] Hassam SL, Mahmood UR, Fahd SL, Salim W, Huma J (2010). Assessment of nutritional status of 1–5 year old children in an urban union council of abbottabad. J Ayub Med Coll Abbottabad..

[27] Hien N, Hoa N (2009). Nutritional status and determinants of malnutrition in children under three years of age in Nghean. Vietnam Pak J Nutr..

[28] Caulfield LE, de Onis M, Blössner M, Black RE (2004). Under nutrition as an underlying cause of child deaths associated with diarrhea, pneumonia, malaria, and measles. Am J Clin Nutr.

[29] Amsalu S, Tigabu Z (2008). Risk factors for severe acute malnutrition in children under the age of five. Ethiop J Health Dev..

[30] USAID (2007). Nutritional status and its determinants in southern Sudan.

[31] Kebede E. Prevalence and determinants of child malnutrition in Gimbi district. 2010;27–39. http://etd.aau.edu.et/handle/123456789/11878. Accessed 14 Feb 2017.

