# Supplementary material for: Magnitude of wasting and underweight among children 6–59 months of age in Sodo Zuria District, South Ethiopia: a community based cross-sectional study
Source: BMC Res Notes. 2018 Nov 3;11:790. doi: 10.1186/s13104-018-3880-x (PMC6215662; doi:10.1186/s13104-018-3880-x)
Supplement: Supplementary file 1 — Additional file 1: Table S1. Socio-demographic characteristics of study participants in Sodo Zuria district, South Ethiopia, June 2017. [file 13104_2018_3880_MOESM1_ESM.docx]

**Additional file 1.Table S1 Socio-demographic characteristics of study participants in Sodo Zuria district, South Ethiopia, June 2017**

| **Variables (n=342)** | | **Frequency** | **Percentage** |
| --- | --- | --- | --- |
| **Maternal Age** | 15-19 | 9 | 2.63 |
|  | 20-29 | 192 | 56.14 |
|  | 30-39 | 121 | 35.38 |
|  | ≥40 | 20 | 5.85 |
| **Marital status** | Currently married | 299 | 87.43 |
|  | Currently unmarried | 43 | 12.57 |
| **Ethnicity** | Wolaita | 336 | 98.25 |
|  | Others* | 6 | 1.75 |
| **Religion** | Protestant | 246 | 71.93 |
|  | Orthodox | 87 | 25.44 |
|  | Others** | 9 | 2.63 |
| **Maternal education** | Can’t read and write | 224 | 65.50 |
|  | Read and write | 7 | 2.05 |
|  | Primary education (1-8) | 90 | 26.31 |
|  | Secondary education (9-12) | 9 | 2.63 |
|  | Above secondary | 12 | 3.51 |
| **Paternal education** | Can’t read and write | 183 | 53.51 |
|  | Read and write | 15 | 4.38 |
|  | Primary education (1-8) | 113 | 33.04 |
|  | Secondary education (9-12) | 11 | 3.22 |
|  | Above secondary | 20 | 5.85 |
| **Maternal occupation** | Housewife | 25 | 7.31 |
|  | Farmer | 239 | 69.88 |
|  | Merchant | 46 | 13.45 |
|  | Others*** | 32 | 9.36 |
| **Paternal occupation** | Farmer | 244 | 71.34 |
|  | Government employee | 25 | 7.31 |
|  | Merchant | 42 | 12.28 |
|  | Other*** | 31 | 9.06 |
| **Monthly Income** | <750 ETB | 258 | 75.44 |
|  | 750-1500 ETB | 68 | 19.88 |
|  | >1500 ETB | 16 | 4.68 |
| **Decision making on income** | Husband | 10 | 2.92 |
|  | Spouse | 38 | 11.11 |
|  | Jointly | 294 | 85.97 |
| **Family size** | ≤5 | 154 | 42.03 |
|  | >5 | 188 | 54.97 |
| **Child sex** | Male | 164 | 47.95 |
|  | Female | 178 | 52.05 |
| **Child age (in months)** | 6-23 | 104 | 30.41 |
|  | 24-59 | 238 | 69.59 |

*widowed, separated **Gurage, Oromo, Amhara ***Muslim, Catholic

****government employee, self employee, student
