# Supplementary material for: Magnitude of wasting and underweight among children 6–59 months of age in Sodo Zuria District, South Ethiopia: a community based cross-sectional study
Source: BMC Res Notes. 2018 Nov 3;11:790. doi: 10.1186/s13104-018-3880-x (PMC6215662; doi:10.1186/s13104-018-3880-x)
Supplement: Supplementary file 2 — Additional file 2: Table S2. Obstetric, child morbidity and other maternal characteristics of study participants in Sodo Zuria district, South Ethiopia, June 2017. [file 13104_2018_3880_MOESM2_ESM.docx]

**Additional file 2: Table S2. Obstetric, child morbidity and other maternal characteristics of study participants in Sodo Zuria district, South Ethiopia, June 2017**

| **Variables (n=342)** | | **Frequency** | **Percentage** |
| --- | --- | --- | --- |
| Place of delivery | Health facility | 272 | 79.53 |
|  | Home | 70 | 20.47 |
| Gestational age at birth | <9 months | 14 | 4.09 |
|  | At 9 month | 229 | 66.96 |
|  | >9 month | 99 | 28.95 |
| Birth weight | <2500 grams | 10 | 2.92 |
|  | 2500-4000 grams | 257 | 75.15 |
|  | >4000 | 75 | 21.93 |
| Birth order | 1 | 66 | 19.30 |
|  | 2-4 | 160 | 46.78 |
|  | >4 | 116 | 33.92 |
| Birth interval from previous | <24 months | 69 | 20.17 |
|  | 24 months | 224 | 65.50 |
|  | >24 months | 49 | 14.33 |
| Still breast feeding | Yes | 202 | 59.07 |
|  | No | 140 | 40.93 |
| Reason for not breast feeding | Maternal health problem | 19 | 13.57 |
|  | Child refusal | 88 | 62.86 |
|  | Pregnancy | 33 | 23.57 |
| Diarrheal morbidity in the last 2 weeks | Yes | 89 | 26.02 |
|  | No | 253 | 73.98 |
| Fever | Yes | 23 | 6.73 |
|  | No | 319 | 93.27 |
| Cough | Yes | 20 | 5.85 |
|  | No | 322 | 94.15 |
| Measles | Yes | 8 | 2.34 |
|  | No | 334 | 97.66 |
| Maternal age at first birth | 15-19 | 48 | 14.03 |
|  | 20-29 | 213 | 62.28 |
|  | 30-39 | 70 | 20.47 |
|  | ≥40 | 11 | 3.22 |
| ANC follow up | Yes | 286 | 83.63 |
|  | No | 56 | 16.37 |
| Additional meal during pregnancy/lactation | Yes | 79 | 23.10 |
|  | No | 263 | 76.90 |
| Family planning method used | Yes | 170 | 49.70 |
|  | No | 172 | 51.30 |
| What type of family planning | Pills | 34 | 20.00 |
|  | Depo-Provera | 131 | 77.06 |
|  | Others* | 5 | 2.94 |

*Norplant, Condom, Intrauterine device
