# Supplementary material for: Magnitude of wasting and underweight among children 6–59 months of age in Sodo Zuria District, South Ethiopia: a community based cross-sectional study
Source: BMC Res Notes. 2018 Nov 3;11:790. doi: 10.1186/s13104-018-3880-x (PMC6215662; doi:10.1186/s13104-018-3880-x)
Supplement: Supplementary file 3 — Additional file 3: Table S3. Child caring practice and environmental health characteristics of study participants in Sodo Zuria district, South Ethiopia, June 2017. [file 13104_2018_3880_MOESM3_ESM.docx]

**Additional file 3: Table S3. Child caring practice and environmental health characteristics of study participants in Sodo Zuria district, South Ethiopia, June 2017**

| **Variables (n=342)** | | **Frequency** | **Percentage** |
| --- | --- | --- | --- |
| Initiation of breast feeding | <1 hour after delivery | 228 | 66.67 |
|  | 1-24 hours | 105 | 30.70 |
|  | After 24 hours | 9 | 2.63 |
| Colostrums feeding | Yes | 334 | 97.66 |
|  | No | 8 | 2.34 |
| Pre lacteal food/liquid | Yes | 41 | 11.99 |
|  | No | 301 | 88.01 |
| Type of pre lacteal food/drink | Water with sugar | 10 | 24.39 |
|  | Butter | 4 | 9.76 |
|  | Cow’s milk | 27 | 65.85 |
| Age at initiation of complementary food | 1-3 months | 32 | 9.36 |
|  | 4-5 months | 77 | 22.51 |
|  | At 6 months | 195 | 57.02 |
|  | After 6 months | 38 | 11.11 |
| Frequency of feeding | <3 times a day | 31 | 9.07 |
|  | 3 times a day | 264 | 77.19 |
|  | >3 times a day | 47 | 13.74 |
| Method of feeding | Hand | 156 | 45.61 |
|  | Spoon | 124 | 36.26 |
|  | Cup | 40 | 11.70 |
|  | Bottle | 22 | 6.43 |
| Variety of food | 2 food items | 235 | 68.71 |
|  | >2 food items | 107 | 31.29 |
| Leftover food in the household | Given to children | 247 | 72.22 |
|  | Given to others | 55 | 16.08 |
|  | Throw away | 40 | 11.70 |
| Ever fed child meat | Yes | 92 | 26.90 |
|  | No | 250 | 73.10 |
| Completed Vaccination | Yes | 327 | 95.61 |
|  | No | 15 | 4.39 |
| Source of drinking water | Protected well/spring | 269 | 78.66 |
|  | Unprotected well/spring | 35 | 10.23 |
|  | Pipe water | 7 | 2.05 |
|  | River | 31 | 9.06 |
| Availability of latrine | Yes | 249 | 72.81 |
|  | No | 93 | 27.19 |
| Materials used for hand washing | Water only | 21 | 6.14 |
|  | Use soap often | 279 | 81.58 |
|  | Use soap always | 42 | 12.28 |
| Waste disposal | Open field | 116 | 33.92 |
|  | In pit | 138 | 40.35 |
|  | Use as compost | 88 | 25.73 |
